# Associations between curriculum-based outdoor education and school-aged children’s physical activity throughout the week

**DOI:** 10.1093/heapro/daag094

**Published:** 2026-07-15

**Authors:** Nanna Wackström, Elina Engberg, Sari Aaltonen, Jenny Ray, Josefine Kailaheimo-Björkqvist, Nina Simonsen

**Affiliations:** Faculty of Medicine, University of Helsinki, PO Box 63, Helsinki FI-00014, Finland; Folkhälsan Research Center, Topeliuksenkatu 20, Helsinki FI-00250, Finland; Faculty of Medicine, University of Helsinki, PO Box 63, Helsinki FI-00014, Finland; Folkhälsan Research Center, Topeliuksenkatu 20, Helsinki FI-00250, Finland; Faculty of Medicine, University of Helsinki, PO Box 63, Helsinki FI-00014, Finland; Institute for Molecular Medicine Finland FIMM, University of Helsinki, PO Box 20, Helsinki FI-00014, Finland; Folkhälsan Research Center, Topeliuksenkatu 20, Helsinki FI-00250, Finland; Faculty of Sport and Health Sciences and Gerontology Research Center, University of Jyväskylä, PO Box 35, Jyväskylä FI-40014, Finland; Folkhälsan Research Center, Topeliuksenkatu 20, Helsinki FI-00250, Finland; Institute of Applied Health Sciences, University of Aberdeen, Foresterhill, Aberdeen AB25 2ZD, United Kingdom; Folkhälsan Research Center, Topeliuksenkatu 20, Helsinki FI-00250, Finland; Department of Public Health, University of Helsinki, PO Box 20, Helsinki FI-00014, Finland

**Keywords:** outdoor learning, outdoor environment, movement integration, health promotion, primary school, children and youth, accelerometry

## Abstract

Integrating curriculum-based outdoor education (OE) into pedagogical practices in primary schools holds potential for health promotion as an alternative to sedentary classroom settings, but it remains understudied. We investigated associations between OE and children’s school- and leisure-time physical activity (PA) throughout the week and possible differences between boys and girls in the associations. This 1-week cross-sectional study conducted in primary schools in Finland (2021–3) used accelerometer-measured PA data from 200 children aged 8–13 years, teacher-reported logbook data on OE, and questionnaire data on sociodemographics. Fifty-two percent of the children had no OE during the study period, whereas the other half had 25–335 minutes (mean 63 minutes). In linear regression models adjusted for sociodemographics, physical education, and season, low to moderate amounts of OE (25–60 minutes during study period), compared to no OE, were associated with 1.2 minutes/hour more moderate to vigorous-intensity PA (MVPA) and 1.8 minutes/hour more total PA (TPA) during school time among all children. The highest amounts of OE (120–335 minutes during study period) were associated with 2.7 minutes/hour more light-intensity PA and 2.8 minutes/hour more TPA on weekend days. Gender moderated some associations; the highest amounts of OE were associated with 3.5 (boys) and 4.8 (girls) minutes/hour more MVPA during school time and, among girls, with 4.2 minutes/hour more TPA during school time and 1.5 minutes/hour more MVPA and 2.2 minutes/hour more TPA on weekdays. These findings suggest that integrating OE into the school day may support PA throughout the week, particularly among girls.

Contribution to Health Promotion

- Schools are important settings for health promotion.
- Curriculum-based outdoor education is positively associated with both school- and leisure-time physical activity, highlighting its potential to promote health and wellbeing in children.
- Outdoor education may be particularly beneficial for girls, who are generally less physically active than boys.
- Participation in outdoor education is positively associated with moderate to vigorous-intensity physical activity, which has well-established health benefits.
- These findings offer insights for shaping curricula and learning environments to promote health.

## Background

Physical activity (PA) is favourably associated with various physical, psychosocial, and cognitive health outcomes in school-aged children, with the benefits being particularly evident for PA of higher intensity, such as moderate to vigorous-intensity PA (MVPA) (Janssen and LeBlanc 2010, Poitras *et al*. 2016, Chaput *et al*. 2020). According to global recommendations, school-aged children should engage in at least one hour of MVPA per day, on average, throughout the week (World Health Organization 2020), but only a minority of children meet the recommendation, with particularly low adherence among girls (Rakić *et al*. 2024). In the context of digitalization and urbanization in modern society, children’s PA is less supported through everyday routines such as active play and active transport, with sedentary behaviours becoming increasingly prevalent (Tremblay *et al*. 2016).

The school day has turned out to be an important setting for PA, particularly for the least physically active children, as nearly half of their daily MVPA occurs during time spent in school (Tammelin *et al*. 2015). As children’s MVPA during the school day has traditionally been concentrated mainly in physical education (PE) lessons and recess time (Carlson *et al*. 2013), creating PA supportive learning environments and integrating activities that encourage PA as part of the everyday, curriculum-based teaching in subjects other than PE might offer children additional opportunities to be physically active during the school day (Trapasso *et al*. 2018, Bentsen *et al*. 2022, Campbell *et al*. 2025). Moreover, schools have considerable potential as health-promoting arenas, such as for PA, since they reach a wide range of children from various backgrounds, and children spend a substantial proportion of their time there (Neil-Sztramko *et al*. 2021, World Health Organization & The United Nations Educational Scientific and Cultural Organization 2021).

Outdoor education (OE) is a curriculum-based approach where teaching is moved outside the school building. The approach provides variety to classroom-based teaching by bringing learning experiences closer to real-life situations and phenomena within authentic environments, thereby supporting children’s learning (Faskunger *et al*. 2018). OE can be conducted in various school subjects and in both natural and built environments, such as schoolyards, forests, other natural landscapes, parks, gardens, and residential areas (Remmen and Iversen 2023). It is often up to the individual teacher to decide what kind of environment is most appropriate in relation to the seasonal context and the academic goals of the lesson (Mygind 2022).

Systematic reviews (Gray *et al*. 2015, James *et al*. 2026) found that children generally are more physically active in outdoor environments compared to indoor environments and emphasized the importance of both unstructured outdoor play and structured outdoor activities in schools. Thus, schools have a crucial role in both promoting PA and providing equal opportunities for all children to engage in outdoor movement and play (World Health Organization 2018, Lee *et al*. 2025). Although the main focus of OE has been on academic learning rather than promoting PA specifically (Mygind 2022), the few existing studies have noticed the potential for increased PA by moving teaching to an outdoor environment. According to altogether five Nordic, British, and German studies using device-based measurements and involving small sample sizes (*n* = 14–48), 7–13-year-old children seem to have a higher average heart rate (Grønningsæter *et al*. 2007), be twice as physically active (Mygind 2007), and engage in more light-intensity PA (LPA) and MVPA (Romar *et al*. 2019) during school time on days with OE compared to regular school days, with some studies also reporting more LPA and MVPA across the whole weekday (Trapasso *et al*. 2018, Romar *et al*. 2019), as well as more MVPA among children with weekly OE compared to those without (Dettweiler *et al*. 2017). Findings from two sub-studies of the Danish TEACHOUT study, involving 361 children aged 9–13 years, suggested that OE may be associated with PA in different ways among boys and girls, generally indicating positive associations with MVPA among boys and LPA among girls (Schneller *et al*. 2017a, Schneller *et al*. 2017b). In light of research consistently indicating that boys engage in more PA and especially more MVPA than girls (Chen *et al*. 2023, Rakić *et al*. 2024), it is warranted to consider whether OE may promote PA and reduce, or instead maintain or increase, these gender differences.

Despite increasing attention to OE in recent years, research on its associations with children’s PA remains scarce and inconclusive. There is little evidence on which PA intensity OE promotes, whether the effects extend beyond school time into leisure time, and how the effects differ between genders. While the effects of many school-based PA interventions tend to be limited to the school day (Guo *et al*. 2025), PA occurring within OE has been suggested to be characterized by self-determined motivation, which in turn is key to engagement, enjoyment, and continued participation in PA (Teixeira *et al*. 2012, Bentsen *et al*. 2022). Whether OE may influence engagement in PA beyond school time remains largely unexplored, with the few existing studies reporting no associations with PA during leisure time on weekdays (Romar *et al*. 2019) or with weekly PA, including both school- and leisure-time on weekdays as well as weekend days (Elsborg *et al*. 2024). However, to the best of our knowledge, no previous study has investigated associations between OE and children’s PA specifically on weekend days, although such associations may exist and warrant further investigation. We investigated associations between curriculum-based OE and PA among school-aged children, focusing on total PA (TPA) and time spent at different intensities of PA (LPA and MVPA) on weekdays, weekend days, during school time, leisure time on weekdays, and as a daily average. We also investigated potential gender differences in the associations.

## Methods

### Study design, setting, and participants

This cross-sectional study, with participants taking part for 1 week, is part of the *LärMiljö (Learning Environment) study: movement, outdoor learning and wellbeing in school* (Simonsen *et al*. 2022). The LärMiljö study is the first of its kind in Finland, aiming to investigate the use of OE in primary schools and its associations with health, wellbeing, PA, learning, and nature connectedness in children. Although OE is not mandated in the Finnish national core curriculum, use of diverse teaching methods and learning environments as well as promotion of physically active lifestyles is emphasized in the current education policy (Finnish National Board of Education 2016, Finnish Government 2024). Data collection took place in school years 2021–22 and 2022–23. Classes in grades 3–6 (children aged 8–13 years) at Swedish-language primary schools in selected municipalities in the Uusimaa, Southwest Finland, and Ostrobothnia regions in Finland were invited to participate. The invitation was sent electronically to the principal of each school, who distributed it to the teachers. Using an electronic registration form, the principal or teacher enrolled the class in the study. The recruitment of classes continued until the goal of over 200 participating children was achieved, with approximately equal numbers participating in and not participating in OE, as recommended for achieving balanced group sizes (Althubaiti 2023). The sample size is similar to those used in previous accelerometer-based research conducted in similar school settings, particularly focusing on outdoor environments and using comparable analytical approaches (e.g. Klinker *et al*. 2014, Pagels *et al*. 2014), and reflects practical and logistical considerations during data collection in the present study.

Information letters designed for children and caregivers were sent electronically and in paper to the teachers of the enrolled classes, whereafter the teacher distributed the materials to all children and their caregivers. All invited teachers, caregivers, and children who consented to participate were included in the study. The researchers visited the classes at their respective schools two times: in the beginning and end of the 1-week study period. Data were collected via electronic questionnaires for children and caregivers, PA measurements, activity diaries, and academic tests for children and an electronic logbook for teachers. The teachers could also, if they liked to, answer a more comprehensive electronic questionnaire on their use of OE.

### Ethical approval and consent to participate

Ethical approval for the LärMiljö study was obtained from the Folkhälsan Research Center’s Ethical Review Board in Humanities (statement FH1/2021_1908). Permission to conduct the study in municipal schools was obtained from each participating municipality. Information letters for schools, children, and caregivers included information on the aim and process of the study, emphasizing that participation is voluntary and that participants can withdraw at any time without consequences. Written informed consent was obtained from all participants (teachers, caregivers, and children). The child’s participation required consent from both the child and the caregiver.

### Measures

#### Physical activity measurement

*Children’s PA* was measured for seven consecutive days using triaxial hip-worn accelerometers (wGT3X+, ActiGraph, LCC, Pensacola, FL, USA). The use of hip-worn accelerometers has been shown to be feasible for measuring PA among school-aged children (Lynch *et al*. 2019). On the same seven days, children completed an activity diary to report wake-up and bedtimes, school times, and accelerometer non-wear times. The accelerometers and activity diaries were distributed by the researchers at the first school visit, and children were given verbal and written instructions on how to use them. The accelerometer was worn on the right hip using an elastic waistband. Children were encouraged to wear the accelerometer consecutively, taking it off only for water-based activities, such as showering, swimming, and sauna.

For two of the participating classes, the study period exceeded a public holiday. One of these two classes had also an extra day off from school the day before the public holiday. For these classes, the study period was 1 or 2 days longer respectively than for the other classes to ensure that the study period included the same number of school days for all classes.

#### Physical activity data processing

ActiLife software version 6.13.4 was used to initialize, download, and process the accelerometer data. The accelerometers were initialized to collect data at a sampling rate of 30 Hertz and to start recording at midnight (12:00 a.m.) on the day of the first research visit and stop recording just before midnight (11:59 p.m.) on the day of the second research visit. Data were processed with an epoch length of 15 seconds and the normal frequency filter, and non-wear time was defined as ≥20 minutes of consecutive zero counts (Migueles *et al*. 2017). The first and last days of recording were excluded from the analyses since the participants started and ended the measurement at different times of the school day, resulting in incomplete days. Participants with at least 600 minutes of accelerometer-measured time per day during waking hours on at least three weekdays and one weekend day were included in the analyses (Migueles *et al*. 2017).

To be able to separate awake time from sleep time and school time from leisure time, filters were created and added to the PA data in ActiLife. Information on wake-up and bedtime was taken from the child’s activity diary. If there were missing data, a general filter (wake-up time 07:00 a.m. and bedtime 11:00 p.m.) was used. Sick days, as well as other absence days, reported by the children in the diary were excluded from the PA data. Information on school times for each class was taken from the teacher’s logbook. Missing data were supplemented using the child’s activity diary or the child’s/caregiver’s questionnaire, as appropriate.

#### Physical activity variables

Cut-points by Evenson *et al*. (2008) were used to create PA variables of different intensities: LPA (26–573 counts/15 seconds) and MVPA (≥574 counts/15 seconds). The PA variables were calculated as the average time (minutes/hour) that the child had spent being physically active at each intensity and in total and were created separately for weekdays, weekend days, and school- and leisure-time on weekdays. The child’s accelerometer-measured time in hours during these different day types and contexts was used as the divisor. Leisure time on weekdays was defined as time outside school time and included the time from waking up until the start of the school day, as well as the time from the end of the school day until bedtime. In addition, a weighted average of daily PA was calculated using the following formula: [(average PA on weekdays in minutes/hour based on days with valid PA data * 5) + (average PA on weekend days in minutes/hour based on days with valid PA data * 2)]/7. The public holiday was classed as a weekend day, as was the extra day off for one of the classes. Moreover, a dichotomous variable was created to indicate whether participants met the PA recommendations, defined as accumulating an average of at least 60 minutes of MVPA per day (World Health Organization 2020).

#### Participation in outdoor education

*The amount of OE during the study period* was reported at class level by the teacher in the logbook for the same days that the children wore accelerometers. For each school day separately, the teacher was asked to report whether the class had engaged in OE and when each OE session started and ended. In line with previous studies (e.g. Schneller *et al*. 2017b, Bølling *et al*. 2021) and the definition of OE used in the LärMiljö study (i.e. *all curriculum-based teaching in subjects other than PE that takes place outside the school building*), outdoor PE sessions were not counted as OE. In addition, OE was restricted to sessions conducted outdoors.

Some teachers had not filled in their logbook on the first and last days of the study period, meaning that there were missing data regarding possible OE sessions on these days. To ensure a comparable number of days of OE data for all classes, the first and last days of the study period were excluded from analyses, similarly to the PA data. Before analyses, one OE session reported by a teacher was changed to a PE session because the content of the session (outdoor sport competition) aligned better with PE. Another teacher from the same school had reported the same session as both OE and PE, and it was changed to PE only. Similarly, one PE session was changed to OE (forest visit for the whole school), which aligned with logbook data from the other teachers at the school. The length of all OE sessions in minutes was then summed for each class. If a child had been away from school (sick or other absence) on a day when the class had engaged in OE, the OE minutes from that day were subtracted from the child’s total OE minutes. In analyses, the OE variable was used both as a continuous and a categorical variable. For the categorical variable, children were divided into three groups based on the distribution of OE minutes that they had participated in during the study period.

#### Covariates

The child’s *gender* and *grade* were reported by the child in the questionnaire. If the child had not answered the questionnaire, information was taken from the caregiver’s questionnaire or consent form, as appropriate. Due to the small number of children (*n* = 4) who reported ‘other/prefer not to answer’ for gender, these responses were treated as missing data and gender was dichotomized into (i) boys and (ii) girls. Grade was dichotomized into (i) 3–4 and (ii) 5–6 for descriptive analyses on class level and regression analyses.

*Accelerometer wear days* were determined by the total number of days for which the child had valid PA data.

*The socioeconomic status (SES)* of the family was determined by the caregivers’ highest level of education. The responding caregiver was asked in the questionnaire to report the highest level of education for themselves and for a possible other caregiver/adult in the household. The highest education level in the household was chosen and categorized into (i) low SES (primary and lower secondary education, or upper secondary education), (ii) middle SES (bachelor’s degree), and (iii) high SES (master’s degree or higher). The categorization was based on the sample distribution to optimize group size comparability.

*The amount of PE during the study period* was reported at class level by the teacher in the logbook for the same days that the children wore accelerometers. For each school day separately, the teacher was asked to report whether the class had engaged in PE and when each PE session started and ended. The length of all PE sessions in minutes was summed for each class, except for possible PE sessions on the first and last days. If a child had been away from school (sick or other absence) on a day when the class had engaged in PE, the PE minutes from that day were subtracted from the child’s total amount of PE minutes.

*Season of participation* was determined by the month in which the class participated in the study. December–February were classified as winter, March–May as spring, and September–November as autumn, corresponding to the official seasonal definitions in Finland (Finnish Meteorological Institute 2025b). In Finland, weather and temperature in winter differ markedly from those in spring and autumn (Finnish Meteorological Institute 2025a). Therefore, the variable was dichotomized into (i) spring or autumn and (ii) winter for regression analyses.

We included the aforementioned covariates since previous studies have suggested that sociodemographic factors like gender, age, and SES are associated with PA in children (Chen *et al*. 2023, Rakić *et al*. 2024, Kokko and Hämylä 2025). PE lessons, in turn, contribute to children’s MVPA during the school day (Carlson *et al*. 2013). Moreover, children’s PA behaviours differ across the seasons in Finland (Kallio *et al*. 2016, Kokko and Hämylä 2025). The number of valid accelerometer wear days influences the reliability and representativeness of the PA data (Migueles *et al*. 2017); thus, these were included to ensure that observed differences in PA reflect true behavioural variations rather than differences in measurement duration.

### Statistical analysis

Descriptive statistics of participating classes and children are presented as frequencies, means with standard deviations (SD), and ranges. Group differences were examined using χ² test, independent-samples *t*-test, one-way ANOVA, or Kruskal–Wallis test as applicable. *Post hoc* tests with Bonferroni correction were conducted for multiple pairwise comparisons (three tests) as applicable, and the adjusted *P-*values are reported. Associations between OE and PA were investigated in linear regression analyses adjusting for the covariates. The regression analyses were conducted separately for each PA intensity and TPA on weekdays, weekend days, during school time, leisure time on weekdays, and as a weighted daily average respectively. OE was used both as a continuous and a categorical variable in the regression analyses. To test for the moderating factor of gender, an interaction term between gender and OE was added to all the fully adjusted models, and if the interaction term turned out to be statistically significant, regression analyses were conducted separately for boys and girls. Statistical significance was set at *P* < 0.05.

## Results

The final study period comprised four weekdays and at least one weekend day. The OE data covered all four weekdays, whereas the PA data covered at least three weekdays and one weekend day.

### Descriptive statistics of participating classes

Altogether, 29 classes from 15 schools across seven municipalities participated in the LärMiljö study. Characteristics of the participating classes are presented in Table 1. Of the total 408 children in all the participating classes, 260 (64%) participated in the study. The participating classes had 0–3 OE sessions during the study period, with half (50%) of the classes having at least one OE session. The amount of OE ranged from 0 to 335 minutes, with an average of 63 minutes, and none of the classes had between 1–24 or 61–119 minutes of OE. Logbook data were lacking for one of the participating classes, meaning that we had no data on OE and PE for that class. Therefore, this class with three participating children was excluded from further analyses.

**Table 1:** Characteristics of the participating classes.

	*N* (%)	Mean (SD)	Range
Participating classes	29		
Participating children per class (*n* = 29)		9.0 (5.2)	1–19
Grade (*n* = 29)			
3–4 (mean age 9.4, SD 0.6 years)	13 (44.8)		
5–6 (mean age 11.4, SD 0.7 years)	16 (55.2)		
Season of participation (*n* = 29)			
Spring	6 (20.7)		
Autumn	16 (55.2)		
Winter	7 (24.1)		
Amount of OE (min) during study period (*n* = 28)		62.9 (106.5)	0–335
0	14 (50.0)		
1–30	2 (7.1)		
31–60	7 (25.0)		
61–90	0 (0.0)		
91–120	1 (3.6)		
121–180	0 (0.0)		
181–240	0 (0.0)		
241–300	2 (7.1)		
>300	2 (7.1)		
Amount of PE (min) during study period (*n* = 28)		101.6 (89.2)	0–370
0	3 (10.7)		
1–60	10 (35.7)		
61–120	8 (28.6)		
121–180	3 (10.7)		
181–240	1 (3.6)		
241–300	2 (7.1)		
>300	1 (3.6)		

SD, standard deviation; OE, outdoor education; PE, physical education; min, minutes; *n*, number of participating classes.

### Descriptive statistics of participating children

Of the 260 children participating in the study, 200 (77%) met the criteria for valid OE and PA data and were included in the analyses. This represented 49% of the total number of possible participants in the participating classes. Included children had on average higher amounts of OE during the study period than children with missing or invalid PA data (86.2 vs. 47.5 minutes, *P* = 0.021). In addition, included children were more likely than children with missing or invalid PA and/or OE data to be in the low SES category (Bonferroni-corrected pairwise comparison against the high SES category, *P* = 0.009), whereas no differences were seen in terms of gender, grade, season, or PE (*P* > 0.088).

Characteristics of included children are presented in Table 2. The mean age was 10.5 years (SD 1.2). Boys were more likely than girls to be in the high SES category (Bonferroni-corrected pairwise comparison against the low SES category, *P* = 0.003). The children had PA data, on average, for 3.9 weekdays (SD 0.3, range 3–4), 2.0 weekend days (SD 0.5, range 1–4), and 5.9 days in total (SD 0.6, range 4–8). The average daily accelerometer-measured time was 849 minutes (SD 42, range 708–948), with an average of 868 minutes on weekdays (SD 45, range 708–976) and 811 minutes on weekend days (SD 70, range 600–1051).

**Table 2:** Characteristics of all children, boys and girls, and by the amount of outdoor education.

	Total	Gender (*n* = 196)			Amount of OE during study period (*n* = 200)			
		Boys	Girls	Group comparison	No OE	Low to moderate amounts of OE (25–60 min)	Highest amounts of OE (120–335 min)	Group comparison
				*P*-value				Overall *P*-value
All participants, *n* (%)	200	81 (41.3)	115 (58.7)		103 (51.5)	46 (23.0)	51 (25.5)	
Gender (*n* = 200)								0.087^a^
Boy, *n* (%)	81 (40.5)				43 (43.0)	23 (51.1)	15 (29.4)	
Girl, *n* (%)	115 (57.5)				57 (57.0)	22 (48.9)	36 (70.6)	
Other/prefer not to answer, *n* (%)	4 (2.0)							
Grade (*n* = 200)				0.307^a^				0.198^a^
3, *n* (%) (mean age 9.0, SD 0.5 years)	42 (21.0)	15 (18.5)	25 (21.7)		26 (25.2)	10 (21.7)	6 (11.8)	
4, *n* (%) (mean age 9.9, SD 0.4 years)	46 (23.0)	17 (21.0)	29 (25.2)		19 (18.4)	11 (23.9)	16 (31.4)	
5, *n* (%) (mean age 10.9, SD 0.4 years)	66 (33.0)	33 (40.7)	32 (27.8)		34 (33.0)	18 (39.1)	14 (27.5)	
6, *n* (%) (mean age 11.9, SD 0.6 years)	46 (23.0)	16 (19.8)	29 (25.2)		24 (23.3)	7 (15.2)	15 (29.4)	
SES (*n* = 193)				**0.004** ^a^				0.112^a^
Low, *n* (%)	55 (28.5)	14 (17.7)	41 (37.3)		24 (24.2)	10 (21.7)	21 (43.8)	
Middle, *n* (%)	44 (22.8)	16 (20.3)	26 (23.6)		23 (23.2)	12 (26.1)	9 (18.8)	
High, *n* (%)	94 (48.7)	49 (62.0)	43 (39.1)		52 (52.5)	24 (52.2)	18 (37.5)	
Season of participation (*n* = 200)				0.279^a^				**<0.001** ^a^
Spring, *n* (%)	23 (11.5)	10 (12.3)	12 (10.4)		13 (12.6)	8 (17.4)	2 (3.9)	
Autumn, *n* (%)	140 (70.0)	60 (74.1)	77 (67.0)		75 (72.8)	16 (34.8)	49 (96.1)	
Winter, *n* (%)	37 (18.5)	11 (13.6)	26 (22.6)		15 (14.6)	22 (47.8)	0 (0.0)	
Amount of PE (min) during study period, mean (SD) (*n* = 200)	120.1 (104.3)	112.5 (101.0)	127.5 (107.2)	0.323^b^	68.0 (58.5)	103.8 (37.6)	239.8 (120.8)	**<0.001** ^c^
Amount of PA (min/h) during study period (*n* = 200)								
School time								
LPA, mean (SD)	18.4 (3.7)	19.2 (3.1)	17.7 (4.0)	**0.004** ^b^	18.4 (3.8)	18.8 (3.7)	17.8 (3.5)	0.445^d^
MVPA, mean (SD)	7.2 (3.4)	8.2 (3.1)	6.5 (3.5)	**<0.001** ^b^	5.4 (2.5)	6.9 (2.8)	11.1 (2.1)	**<0.001** ^d^
TPA, mean (SD)	25.6 (5.1)	27.4 (4.0)	24.3 (5.4)	**<0.001** ^b^	23.8 (5.0)	25.7 (4.7)	28.9 (3.8)	**<0.001** ^d^
Leisure time on weekdays								
LPA, mean (SD)	17.7 (3.2)	17.1 (3.1)	18.2 (3.2)	**0.027** ^b^	17.4 (3.0)	16.8 (3.1)	19.2 (3.2)	**<0.001** ^d^
MVPA, mean (SD)	4.4 (2.1)	4.6 (2.2)	4.1 (1.9)	0.101^b^	4.4 (2.2)	4.2 (2.1)	4.4 (1.9)	0.797^d^
TPA, mean (SD)	22.1 (4.5)	21.7 (4.7)	22.3 (4.3)	0.407^b^	21.8 (4.4)	21.0 (4.3)	23.6 (4.6)	**0.014** ^d^
Weekdays								
LPA, mean (SD)	18.0 (2.8)	17.9 (2.5)	18.1 (3.0)	0.702^b^	17.8 (2.7)	17.6 (2.7)	18.8 (2.7)	0.054^d^
MVPA, mean (SD)	5.3 (2.0)	5.8 (1.9)	4.9 (1.9)	**0.002** ^b^	4.8 (1.9)	5.2 (2.1)	6.6 (1.6)	**<0.001** ^d^
TPA, mean (SD)	23.3 (3.9)	23.7 (3.7)	23.0 (4.1)	0.194^b^	22.5 (3.8)	22.8 (3.7)	25.3 (3.8)	**<0.001** ^d^
Weekend days								
LPA, mean (SD)	17.3 (3.8)	16.8 (3.7)	17.6 (3.8)	0.192^b^	16.8 (3.6)	16.2 (3.9)	19.3 (3.3)	**<0.001** ^d^
MVPA, mean (SD)	4.1 (2.4)	4.6 (2.7)	3.6 (2.0)	**0.005** ^b^	4.0 (2.2)	3.6 (2.2)	4.7 (2.7)	0.054^d^
TPA, mean (SD)	21.4 (5.2)	21.5 (5.4)	21.2 (5.1)	0.711^b^	20.8 (4.9)	19.8 (5.0)	24.0 (5.0)	**<0.001** ^d^
Daily weighted average								
LPA, mean (SD)	17.8 (2.7)	17.6 (2.5)	17.9 (2.9)	0.428^b^	17.5 (2.7)	17.2 (2.8)	18.9 (2.5)	**0.002** ^d^
MVPA, mean (SD)	5.0 (1.9)	5.5 (1.9)	4.6 (1.7)	**<0.001** ^b^	4.6 (1.7)	4.7 (2.0)	6.0 (1.7)	**<0.001** ^d^
TPA, mean (SD)	22.8 (3.8)	23.1 (3.6)	22.5 (4.0)	0.276^b^	22.0 (3.6)	21.9 (3.7)	25.0 (3.6)	**<0.001** ^d^
Meeting PA recommendations (*n* = 200)				**<0.001** ^a^				**<0.001** ^a^
Yes, *n* (%)	120 (60.0)	60 (74.1)	56 (48.7)		52 (50.5)	24 (52.2)	44 (86.3)	
No, *n* (%)	80 (40.0)	21 (25.1)	59 (51.3)		51 (49.5)	22 (47.8)	7 (13.7)	

Statistically significant results (*P* < 0.05) are in bold.

OE, outdoor education; SES, socioeconomic status (categories of the highest educational level of the family: low, primary and lower secondary education, or upper secondary education; middle, bachelor’s degree; and high, master’s degree or higher); PE, physical education; SD, standard deviation; LPA, light physical activity; MVPA, moderate to vigorous physical activity; TPA, total physical activity; *n*, number of participating children; min, minutes; min/h, minutes/hour; meeting PA recommendations, accumulating an average of at least 60 minutes of MVPA per day.

^a^χ² test.

^b^Independent-samples *t*-test.

^c^Kruskal–Wallis test.

^d^One-way ANOVA.

Approximately half (52%) of the children had no OE during the study period, whereas 23% had 25–60 minutes of OE (referred to as *low to moderate amounts of OE*) and 26% had 120–335 minutes of OE (referred to as the *highest amounts of OE*). Differences between these groups were seen in season and PE: those who had the highest amounts of OE had been most likely to participate in the study in autumn and had the highest amounts of PE as well. Differences in PA were seen according to gender and the amount of OE, most pronounced during school time and generally in favour of boys and those who had the highest amounts of OE, with similar patterns observed in the proportion meeting the PA recommendations.

### Associations between outdoor education and physical activity

**Figure 1:**
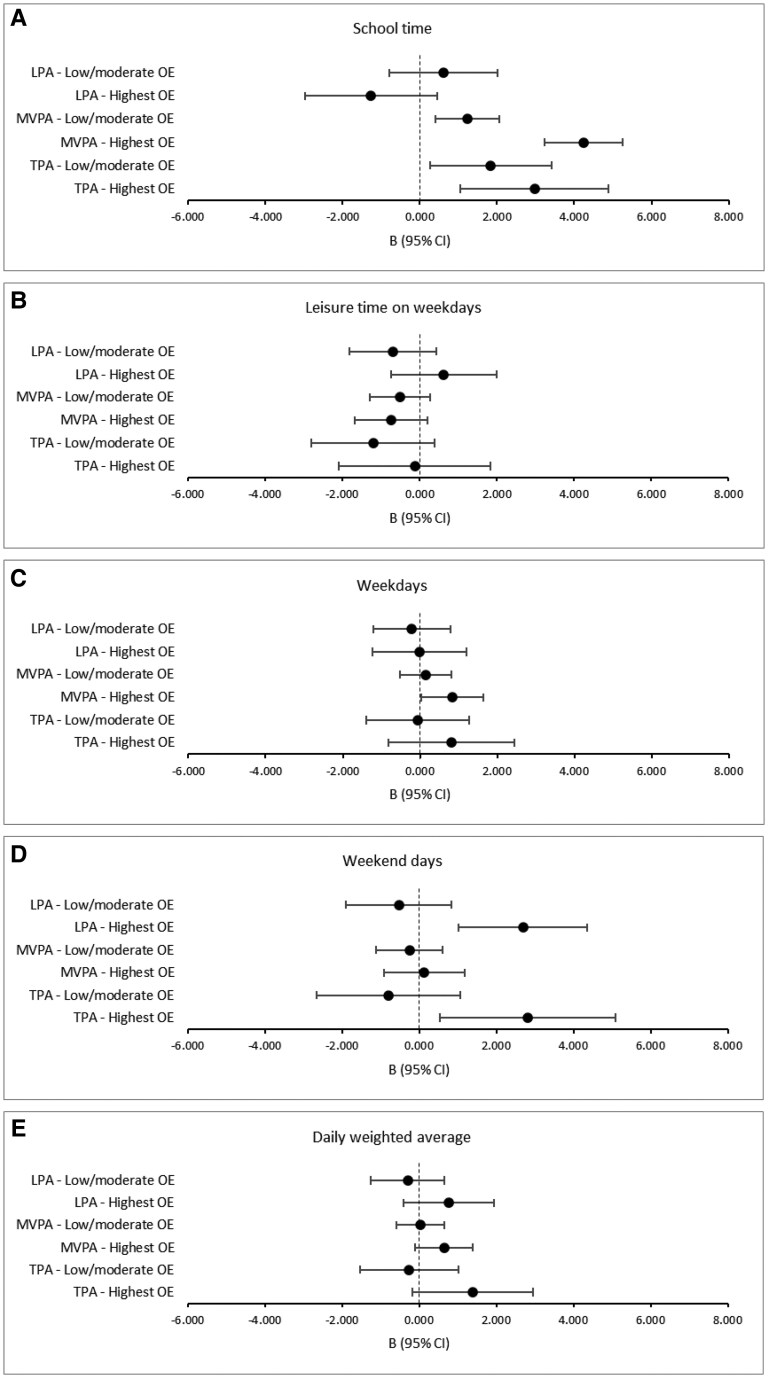
Linear regression results of associations between OE and PA: total sample. Forest plots showing the unstandardized regression coefficient (B) and 95% CI for the associations between OE and PA in the total sample. The dependent variable represents minutes/hour of LPA, MVPA, and TPA across (a) school time, (b) leisure time on weekdays, (c) weekdays, (d) weekend days, and as a (e) daily weighted average. The independent variable represents the amount of OE in minutes during the study period divided into three categories: no OE (reference group), low/moderate amounts of OE (low/moderate OE; 25–60 minutes), and the highest amounts of OE (highest OE; 120–335 minutes). Models were adjusted for gender, grade, accelerometer wear days, PE during study period, season of participation, and SES. Missing data were handled using listwise deletion; analyses included participants with complete data (*n* = 189).

**Figure 2:**
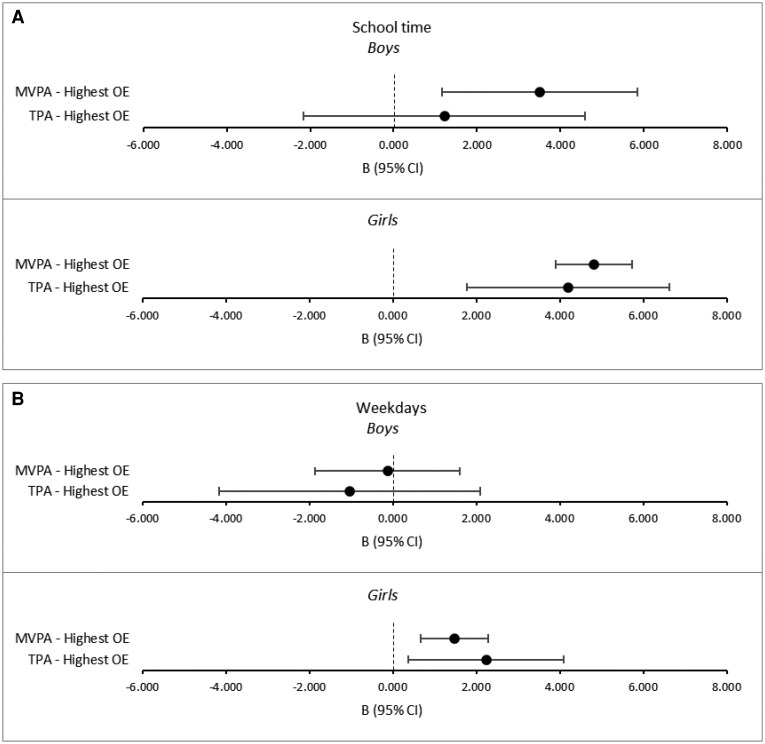
Linear regression results of associations between OE and PA in boys and girls. Forest plots showing the unstandardized regression coefficient (B) and 95% CI for the associations between OE and PA among boys and girls separately. Results are presented only for models where the moderator analysis suggested gender differences. The dependent variable represents minutes/hour of MVPA and TPA across (a) school time and (b) weekdays. The independent variable represents the amount of OE in minutes during the study period divided into three categories: no OE (reference group), low/moderate amounts of OE (low/moderate OE; 25–60 minutes), and the highest amounts of OE (highest OE; 120–335 minutes). Models were adjusted for grade, accelerometer wear days, PE during study period, season of participation, and SES. Missing data were handled using listwise deletion; analyses included participants with complete data (boys *n* = 79, girls *n* = 110).

As shown in Fig. 1 (see also Supplementary Tables S1–S3), the fully adjusted models with OE as a categorical variable showed that in the total sample, low to moderate amounts of OE, compared to no OE, were associated with 1.2 minutes/hour more MVPA [95% confidence interval (CI) 0.4–2.1] and 1.8 minutes/hour more TPA (95% CI 0.3–3.4) during school time. The highest amounts of OE, compared to no OE, were associated with 4.2 minutes/hour more MVPA (95% CI 3.2–5.2) and 3.0 minutes/hour more TPA (95% CI 1.1–4.9) (with a non-significant tendency towards less LPA) during school time, 0.8 minutes/hour more MVPA (95% CI 0.0–1.6) on weekdays, and 2.7 minutes/hour more LPA (95% CI 1.0–4.3) and 2.8 minutes/hour more TPA (95% CI 0.5–5.1) on weekend days. There were significant interactions between gender and the highest amounts of OE for MVPA and TPA during school time and on weekdays. According to gender-specific analyses (Fig. 2; see also Supplementary Tables S1–S3), the highest amounts of OE, compared to no OE, were associated with 3.5 minutes/hour more MVPA (95% CI 1.2–5.8) during school time among boys and 4.8 minutes/hour more MVPA (95% CI 3.9–5.7) during school time among girls. Furthermore, among girls only, the highest amounts of OE, compared to no OE, were associated with 4.2 minutes/hour more TPA (95% CI 1.8–6.6) during school time and 1.5 minutes/hour more MVPA (95% CI 0.7–2.3) and 2.2 minutes/hour more TPA (95% CI 0.4–4.1) on weekdays. The highest proportion of explained variance was observed for MVPA during school time among girls who had the highest amounts of OE, where the model explained 80% of the variance. No associations between OE and PA during leisure time on weekdays were observed.

Similar results were found with OE as a continuous variable (Supplementary Figures S1 and S2), with additional associations found between higher amounts of OE and more MVPA as a daily average and less LPA during school time.

## Discussion

In this study, we investigated associations between curriculum-based OE and children’s school- and leisure-time PA throughout the week, as well as possible differences between boys and girls in the associations. The main results revealed positive associations between OE and children’s PA during both school- and leisure-time, with the associations being particularly pronounced during school time and among girls.

The classes participating in our study had on average 63 minutes of OE during the study period. This is less than in the Danish TEACHOUT study, where an average of 300 weekly minutes of OE throughout a whole school year was the goal for the OE classes and contributed to increased daily MVPA among boys (Schneller *et al*. 2017a, 2017b). In our study, only two of the participating classes had over 300 minutes of OE during the study period. However, our results indicate that even smaller amounts of OE can be positively associated with children’s MVPA and TPA during school time. According to the associations we found for both boys and girls, participating in low to moderate amounts of OE (i.e. 25–60 minutes during the study period) would, during a 5-hour school day, translate to an additional 6 minutes of MVPA and 9 minutes of TPA on average. Nevertheless, the associations seem to be even more pronounced for higher amounts of OE, particularly among girls. Participation in the highest amounts of OE (i.e. 120–335 minutes during the study period) would, during a 5-hour school day, translate to an additional 17.5 minutes of MVPA on average for boys and an additional 24.0 minutes of MVPA and 20.5 minutes of TPA on average for girls.

Overall, our findings correspond to previous research indicating positive associations between OE and children’s PA during school time (Grønningsæter *et al*. 2007, Mygind 2007, Romar *et al*. 2019, Elsborg *et al*. 2024). Outdoor environments may offer more space and varied movement opportunities than indoor settings. OE often also includes interaction with peers and the environment, which may encourage more PA than traditional classroom settings (Bentsen *et al*. 2022, Campbell *et al*. 2025). However, there are variations in the PA intensities associated with OE across studies, which may be due to differences in study designs, methodologies, and the implementation of OE. In addition to MVPA, previous studies have found that children, on average, engage in more LPA during school time in relation to OE (Romar *et al*. 2019, Elsborg *et al*. 2024), a finding that contrasts with our results regarding LPA. More specifically, Schneller *et al*. (2017b) found that boys tend to engage in more MVPA and girls in more LPA during OE sessions compared to classroom teaching.

We did not observe associations between OE and children’s PA during leisure time on weekdays, and a similar tendency was seen by Romar *et al*. (2019). Hence, this finding does not support the compensation theory, according to which increased PA during one part of the day (e.g. school time) would be followed by decreased PA during another part of the day (e.g. leisure time) (Beck *et al*. 2022). According to our results, on a weekly level, OE does not seem to significantly influence how physically active children are after and before school time on weekdays. Instead, children seem to engage in just as much PA regardless of whether they have OE during school time or not. However, in our study, girls who participated in the highest amounts of OE engaged in more MVPA and TPA overall on weekdays, when both school- and leisure-time on weekdays were considered. This partially aligns with previous studies, which did not examine gender differences in this association but have shown that, on weekdays, children on average engage in more MVPA (Romar *et al*. 2019) and LPA (Trapasso *et al*. 2018, Romar *et al*. 2019) when OE is implemented during the school day. As OE was not associated with leisure-time PA on weekdays specifically in our study, it seems that the positive association between OE and girls’ MVPA and TPA during school time is reflected also in their MVPA and TPA overall on weekdays. This highlights the importance of OE to support girls’ PA on weekdays.

While previous studies have compared children’s PA on school days with OE and weekend days (Schneller *et al*. 2017b, Trapasso *et al*. 2018), our study is, to the best of our knowledge, the first to investigate how OE *per se* is associated with children’s PA on weekend days. We found that participation in the highest amounts of OE was associated with more LPA and TPA on weekend days among both boys and girls. This finding was somewhat unexpected, as a recent meta-analysis (Guo *et al*. 2025) suggested that school-based PA interventions often succeed in increasing children’s PA only during school time. However, OE is likely to differ from traditional school-based PA interventions, for example, in that it is part of the normal curriculum-based teaching and primarily focuses on academic learning. By emphasizing pupil autonomy, competence, and social interaction in physically active learning activities, PA during OE may, at its best, be driven by self-determined motivation, which may in turn promote motivational processes that extend beyond the school context (Teixeira *et al*. 2012, Bentsen *et al*. 2022). In addition, OE may contribute to the development of competencies and skills related to the outdoors. As studies suggest that children’s nature connectedness increases with OE in natural environments (Pirchio *et al*. 2021), they might become more eager to spend time outdoors, also during weekends. For example, in focus group interviews conducted by Trapasso *et al*. (2018), children reported replicating activities from the OE sessions at home and engaging in more PA together with their families than before as a result of OE sessions in the forest. As we were unable to account for potential additional confounding factors during the weekend, an important direction for future research is to investigate whether the observed associations reflect the influence of OE itself or other factors related to PA during weekends.

We observed that participation in higher amounts of OE was associated with more MVPA as a daily average for the whole week. Although the magnitude of the association was modest, this is a promising finding from a public health perspective, since only a small proportion of school-aged children meet the current recommendation of at least 1 hour of daily MVPA (Rakić *et al*. 2024, Kokko and Hämylä 2025). In contrast, OE did not show clear associations with LPA and TPA as a daily average for the whole week. Despite some methodological differences, Elsborg *et al*. (2024) partially support our results, indicating positive associations between OE and children’s LPA and MVPA during school time, but no associations with overall LPA and MVPA for the whole week. The higher amounts of LPA and TPA in specific contexts, with which higher amounts of OE were associated in our study, might have been too small to be reflected in the daily average of LPA and TPA for the whole week. Furthermore, in contrast to previous research, we observed tendencies indicating an association between higher amounts of OE and less LPA during school time, which also affects the daily average.

Our study contributes crucial evidence regarding gender differences in the associations between OE and PA. Previously, the TEACHOUT study found associations between weekly participation in OE and increased daily MVPA among boys, whereas, among girls, more LPA on school days with OE compared to regular school days was observed (Schneller *et al*. 2017a, 2017b). Moreover, based on observational data in a sample of 11 children, Fiskum and Jacobsen (2012) found more pronounced differences in PA between indoor and outdoor educational settings among boys. Contrary to these findings, our results suggest that girls may benefit even more than boys from participating in OE in terms of PA. Similarly, potentially supporting our results, Romar *et al*. (2019) suggested that the least physically active children might be those who benefit the most from participating in OE in terms of PA. Girls are generally less physically active than boys (Chen *et al*. 2023, Rakić *et al*. 2024, Kokko and Hämylä 2025), which was also the case in our sample, with 74% of the boys and 49% of the girls meeting the PA recommendations. A systematic review (Duffey *et al*. 2021) including 10*–*19-year-olds suggested that such gender differences are shaped by individual, social, and environmental factors, including perceived competence, confidence, body image, gender norms, peer and family support, safety, access, and the often competitive or sport-centred structure of many PA opportunities. From this perspective, OE may offer a potentially more equitable route to PA because it is integrated within compulsory school time and ordinary curriculum-based activities, rather than relying on voluntary participation in sport or extracurricular PA. The positive associations between OE and PA especially among girls in our study are promising, since earlier findings (Schneller *et al*. 2017a, 2017b) have given rise to a discussion about whether OE may even increase the already existing differences in PA between boys and girls. This may also be particularly relevant from a long-term perspective, as evidence suggests that engagement in diverse PA during adolescence is important for leisure-time PA in adulthood, especially among females (Mäkelä *et al*. 2017). Thus, providing varied opportunities for PA, for example, through OE, may have health benefits that extend beyond childhood.

Differences in how OE is implemented may influence its associations with PA, potentially explaining variation in results across studies. The teacher’s choice of location and teaching methods during OE may influence pupils’ opportunities and motivation to engage in PA (Bentsen *et al*. 2022, Mygind 2022, Campbell *et al*. 2025). For example, children seem to be more physically active when OE is conducted in natural environments, such as forests and parks, compared to other kinds of environments (Bølling *et al*. 2021). Also, the extent to which teachers integrate active transportation and other movement-based activities into OE can vary across teachers (Bentsen *et al*. 2022, Mygind 2022). These kinds of factors may explain why our findings on gender differences differed from those in previous studies (Fiskum and Jacobsen 2012, Schneller *et al*. 2017a, 2017b). As discussed previously by Schneller *et al*. (2017a, 2017b), the less physically active children, who from a gender perspective, are typically girls, may generally benefit more from structured activities led by the teacher in a supportive context to be physically active, while boys may be physically active more spontaneously when they see an opportunity for it. Also, there are studies indicating that exposure to green spaces, for example, in the schoolyard, can increase PA to a greater degree among girls than boys (van Dijk-Wesselius *et al*. 2018). However, Bølling *et al*. (2021) did not find any gender differences in how children’s PA varied across different kinds of environments in which the OE sessions were conducted. The influence of both the physical and social environment, as well as the pedagogical approach and content of OE, is a factor worth considering further when investigating gender differences in the associations between OE and PA.

Our findings have important implications for both practice and policy. By integrating OE into teaching, schools and teachers can offer children additional opportunities for PA during the school day, thereby also supporting their health and wellbeing, without compromising academic learning goals. Strengthening the role of OE within the curriculum and its learning outcomes, enhancing teachers’ competence through targeted training and resources, and ensuring access to appropriate outdoor learning environments near schools may further support its implementation (Bentsen *et al*. 2010, Skaugen and Fiskum 2015, van Dijk-Wesselius *et al*. 2020). Moreover, this corresponds with global recommendations for health-promoting schools, which identify curriculum content and pedagogy as key components in promoting PA (World Health Organization and The United Nations Educational Scientific and Cultural Organization 2021). The Finnish context provides a relevant example, as the national core curriculum emphasizes the role of schools in promoting PA (Finnish National Board of Education 2016), and recent legislative changes further underscore this responsibility (Finnish Government 2024), suggesting the relevance of approaches such as OE.

### Strengths and limitations

The topic of this study is one of its main strengths, as associations between OE and children’s school- and leisure-time PA measured by accelerometers have been investigated in only a few previous studies. The study is one of the first to investigate possible gender differences in the associations and the first to investigate associations with PA on weekend days. Another strength is the utilization of device-measured PA data from different day types and contexts throughout the week. Moreover, the study features one of the larger sample sizes among studies of this topic with children from several municipalities and schools, both of which varied in size. Participants included in the analyses were relatively evenly distributed based on gender, grade, and participation versus non-participation in OE. Controlling for the seasonal context is relatively unique to studies on OE and PA conducted across multiple seasons and adds value to the findings.

However, the cross-sectional study design limits the conclusions regarding causality in the observed associations. The sample may be somewhat selected, for example, with participants with an interest in PA, as a relatively high proportion (60%) met the PA recommendations, although methodological choices in data processing may have influenced these estimates (Chen *et al*. 2023). The sample also showed a higher proportion of highly educated parents compared to the general Finnish population aged 30–55 years (Statistics Finland 2025), which may affect the generalisability of the results. Furthermore, children included in the analyses had slightly different characteristics than children with missing or invalid accelerometer-measured PA data and/or teacher-reported OE data. For example, included children had, on average, more OE during the study period than children with missing or invalid PA data. One potential explanation may be related to teacher engagement, as teachers with a particular interest in the study and OE may have been more likely to motivate pupils to participate in and complete the study. However, as no information on the reasons for missing or invalid data was available, this remains speculative. Nevertheless, as approximately half of the participants included in the analyses had no OE, the sample still represented a relatively broad range of OE exposure. Although the sample size was comparable to previous studies, group sizes were relatively small in the gender-specific regression analyses when OE was treated as a categorical variable, with only 15 boys in the group that participated in the highest amounts of OE. This should be considered when interpreting the results, particularly given the absence of an *a priori* power calculation. However, we observed similar results when we treated OE as a continuous variable, suggesting robustness of the findings. Moreover, the choice of epoch length and non-wear time definition, as well as the use of a general filter to replace missing data on child-reported waking hours in processing the accelerometer-measured PA data, could potentially have affected the results. We processed the PA data with an epoch length of 15 seconds, which is used in most previous studies investigating associations between OE and device-measured PA (e.g. Schneller *et al*. 2017a, 2017b, Romar *et al*. 2019, Bølling *et al*. 2021). However, lower epoch lengths (e.g. 1–10 seconds) are also commonly used among school-aged children (Migueles *et al*. 2017). As we investigated the associations on a weekly level and only accounted for the time spent in OE, rather than the specific activities performed during these sessions, future studies could include more detailed day- and session-specific analyses to explore differences in PA between days with and without OE, while also considering contextual aspects of OE.

## Conclusion

This study contributes crucial evidence on how curriculum-based OE is associated with children’s school- and leisure-time PA throughout the week. Low to moderate amounts of OE (25–60 minutes during the study period) appeared sufficient for modestly higher MVPA and TPA during school time, with more pronounced associations observed at higher amounts of OE (120–335 minutes during the study period), particularly among girls. The associations may extend beyond school time, with higher amounts of OE associated with more LPA and TPA on weekend days. Among girls, higher amounts of OE were also associated with more MVPA and TPA on weekdays. These findings suggest that integrating OE into the school day may support children’s PA, an important determinant of health and wellbeing. Moreover, the finding that girls may benefit even more than boys from participating in OE is noteworthy, given that girls are generally less physically active than boys, potentially due to individual, social, and environmental barriers identified in previous studies. OE may offer a more equitable route to PA than many other PA opportunities by being integrated into everyday school activities, suggesting its relevance for school practice and education policy.

## Supplementary Material

daag094_Supplementary_Data

## Data Availability

The datasets used and analysed during the current study are not publicly available due to the privacy of participants but are available from the corresponding author on reasonable request.
