# Supplementary material for: Associations between curriculum-based outdoor education and school-aged children’s physical activity throughout the week
Source: Health Promot Int. 2026 Jul 15;41(4):daag094. doi: 10.1093/heapro/daag094 (PMC13394712; doi:10.1093/heapro/daag094)
Supplement: daag094_Supplementary_Data [file daag094_supplementary_data.zip › Table S1.docx]

| **Table S1.** Linear regression results of associations between outdoor education and **light physical activity.** | | | | | | | | | | | | | | | | | | | | | | | | | | |
| --- | --- | --- | --- | --- | --- | --- | --- | --- | --- | --- | --- | --- | --- | --- | --- | --- | --- | --- | --- | --- | --- | --- | --- | --- | --- | --- |
|  | Model | **Light physical activity during study period (min/h)** | | | | | | | | | | | | | | | | | | | | | | | | |
|  |  | **School time** | | | | | **Leisure time on weekdays** | | | | | **Weekdays** | | | | | **Weekend days** | | | | | **Daily weighted average** | | | | |
|  |  | B | 95% CI | | p-value | R^2^/adj. R^2^ | B | 95% CI | | p-value | R^2^/adj. R^2^ | B | 95% CI | | p-value | R^2^/adj. R^2^ | B | 95% CI | | p-value | R^2^/adj. R^2^ | B | 95% CI | | p-value | R^2^/adj. R^2^ |
|  |  |  | Lowest | Highest |  |  |  | Lowest | Highest |  |  |  | Lowest | Highest |  |  |  | Lowest | Highest |  |  |  | Lowest | Highest |  |  |
| Amount of OE (min) during study period; **total sample** |  |  |  |  |  |  |  |  |  |  |  |  |  |  |  |  |  |  |  |  |  |  |  |  |  |  |
|  | 1 | -0.003 | -0.007 | 0.001 | 0.122 | 0.012 | 0.006 | 0.003 | 0.010 | **<0.001** | 0.065 | 0.003 | 0.000 | 0.006 | **0.043** | 0.020 | 0.009 | 0.005 | 0.013 | **<0.001** | 0.092 | 0.005 | 0.002 | 0.008 | **0.001** | 0.050 |
|  | 2 | -0.002 | -0.006 | 0.002 | 0.272 | 0.081 | 0.006 | 0.003 | 0.010 | **<0.001** | 0.168 | 0.003 | 0.000 | 0.006 | **0.022** | 0.126 | 0.009 | 0.006 | 0.013 | **<0.001** | 0.167 | 0.005 | 0.002 | 0.008 | **<0.001** | 0.171 |
|  | 3 | -0.007 | -0.012 | -0.001 | **0.014** | 0.103 | 0.002 | -0.002 | 0.007 | 0.305 | 0.193 | -0.001 | -0.005 | 0.003 | 0.708 | 0.164 | 0.009 | 0.003 | 0.014 | **0.001** | 0.168 | 0.002 | -0.002 | 0.006 | 0.301 | 0.195 |
|  | 4 | -0.006 | -0.012 | -0.001 | **0.025** | 0.087 | 0.002 | -0.002 | 0.007 | 0.292 | 0.193 | -0.001 | -0.005 | 0.003 | 0.786 | 0.160 | 0.010 | 0.004 | 0.015 | **<0.001** | 0.169 | 0.002 | -0.001 | 0.006 | 0.220 | 0.194 |
| Interaction term |  | 0.004 | -0.005 | 0.013 | 0.415 | 0.086 | 0.005 | -0.002 | 0.012 | 0.180 | 0.196 | 0.005 | -0.002 | 0.011 | 0.140 | 0.165 | 0.002 | -0.007 | 0.011 | 0.621 | 0.165 | 0.004 | -0.002 | 0.010 | 0.197 | 0.197 |
| Amount of OE (min) during study period (ref. 0 min OE); **total sample** |  |  |  |  |  |  |  |  |  |  |  |  |  |  |  |  |  |  |  |  |  |  |  |  |  |  |
| Low to moderate amounts of OE (25–60 min) | 1 | 0.398 | -0.901 | 1.697 | 0.546 | 0.008 | -0.529 | -1.607 | 0.549 | 0.334 | 0.079 | -0.170 | -1.124 | 0.785 | 0.727 | 0.029 | -0.609 | -1.967 | 0.649 | 0.341 | 0.101 | -0.295 | -1.222 | 0.632 | 0.531 | 0.062 |
|  | 2 | 0.404 | -0.868 | 1.677 | 0.532 | 0.074 | -0.690 | -1.729 | 0.348 | 0.192 | 0.178 | -0.267 | -1.192 | 0.657 | 0.569 | 0.130 | -0.737 | -1.972 | 0.497 | 0.240 | 0.172 | -0.402 | -1.289 | 0.486 | 0.373 | 0.179 |
|  | 3 | 0.640 | -0.743 | 2.022 | 0.362 | 0.091 | -0.700 | -1.825 | 0.424 | 0.221 | 0.198 | -0.202 | -1.198 | 0.794 | 0.690 | 0.159 | -0.530 | -1.887 | 0.828 | 0.443 | 0.166 | -0.295 | -1.258 | 0.667 | 0.546 | 0.195 |
|  | 4 | 0.604 | -0.794 | 2.002 | 0.395 | 0.076 | -0.699 | -1.823 | 0.424 | 0.221 | 0.197 | -0.211 | -1.204 | 0.783 | 0.676 | 0.156 | -0.536 | -1.898 | 0.826 | 0.438 | 0.167 | -0.304 | -1.262 | 0.655 | 0.533 | 0.194 |
| Interaction term |  | 1.490 | -1.118 | 4.099 | 0.261 | 0.077 | -0.379 | -2.474 | 1.717 | 0.199 | 0.199 | 0.378 | -1.474 | 2.229 | 0.688 | 0.159 | 0.416 | -2.138 | 2.971 | 0.748 | 0.159 | 0.389 | -1.401 | 2.178 | 0.669 | 0.194 |
| Highest amounts of OE (120–335 min) | 1 | -0.554 | -1.808 | 0.700 | 0.384 | 0.008 | 1.832 | 0.791 | 2.872 | **<0.001** | 0.079 | 1.013 | 0.092 | 1.935 | **0.031** | 0.029 | 2.494 | 1.279 | 3.709 | **<0.001** | 0.101 | 1.436 | 0.541 | 2.331 | **0.002** | 0.062 |
|  | 2 | -0.292 | -1.516 | 0.932 | 0.639 | 0.074 | 1.745 | 0.746 | 2.744 | **<0.001** | 0.178 | 1.054 | 0.164 | 1.943 | **0.021** | 0.130 | 2.575 | 1.387 | 3.763 | **<0.001** | 0.172 | 1.488 | 0.635 | 2.342 | **<0.001** | 0.179 |
|  | 3 | -1.361 | -3.017 | 0.296 | 0.107 | 0.091 | 0.593 | -0.755 | 1.941 | 0.386 | 0.198 | -0.066 | -1.259 | 1.128 | 0.914 | 0.159 | 2.418 | 0.791 | 4.045 | **0.004** | 0.166 | 0.644 | -0.510 | 1.798 | 0.272 | 0.195 |
|  | 4 | -1.267 | -2.977 | 0.443 | 0.146 | 0.076 | 0.622 | -0.753 | 1.997 | 0.373 | 0.197 | -0.021 | -1.237 | 1.195 | 0.973 | 0.156 | 2.681 | 1.015 | 4.347 | **0.002** | 0.167 | 0.751 | -0.422 | 1.924 | 0.208 | 0.194 |
| Interaction term |  | 1.679 | -1.070 | 4.428 | 0.230 | 0.077 | 1.442 | -0.766 | 3.651 | 0.199 | 0.199 | 1.605 | -0.346 | 3.557 | 0.106 | 0.159 | 0.672 | -2.021 | 3.364 | 0.623 | 0.159 | 1.338 | -0.548 | 3.225 | 0.163 | 0.194 |
| Results from analyses with OE as a continuous and a categorical variable are presented separately. Moderator analysis suggested no gender differences, i.e., the interaction term between gender and OE was not statistically significant in the fully adjusted models; therefore, analyses were not conducted separately for boys and girls. Model 1 is unadjusted; Model 2 is adjusted for gender, grade and accelerometer wear days; Model 3 is adjusted for gender, grade, accelerometer wear days, physical education during study period and season of participation; Model 4 is adjusted for gender, grade, accelerometer wear days, physical education during study period, season of participation and socioeconomic status. Missing data were handled using listwise deletion; for each model, participants with complete data on all variables in that specific model were included (n=189–200). R^2^ value is reported for the unadjusted model (1) and adjusted R^2^ value for the adjusted models (3–4). Statistically significant results (p<0.05) are in bold. OE = outdoor education, B = Unstandardised regression coefficient, CI = Confidence Interval, R^2^ = Coefficient of determination, min = minutes, min/h = minutes/hour. | | | | | | | | | | | | | | | | | | | | | | | | | | |
