# Supplementary material for: Associations between curriculum-based outdoor education and school-aged children’s physical activity throughout the week
Source: Health Promot Int. 2026 Jul 15;41(4):daag094. doi: 10.1093/heapro/daag094 (PMC13394712; doi:10.1093/heapro/daag094)
Supplement: daag094_Supplementary_Data [file daag094_supplementary_data.zip › Table S2.docx]

| **Table S2.** Linear regression results of associations between outdoor education and **moderate to vigorous physical activity.** | | | | | | | | | | | | | | | | | | | | | | | | | | |
| --- | --- | --- | --- | --- | --- | --- | --- | --- | --- | --- | --- | --- | --- | --- | --- | --- | --- | --- | --- | --- | --- | --- | --- | --- | --- | --- |
|  | Model | **Moderate to vigorous physical activity during study period (min/h)** | | | | | | | | | | | | | | | | | | | | | | | | |
|  |  | **School time** | | | | | **Leisure time on weekdays** | | | | | **Weekdays** | | | | | **Weekend days** | | | | | **Daily weighted average** | | | | |
|  |  | B | 95% CI | | p-value | R^2^/adj. R^2^ | B | 95% CI | | p-value | R^2^/adj. R^2^ | B | 95% CI | | p-value | R^2^/adj. R^2^ | B | 95% CI | | p-value | R^2^/adj. R^2^ | B | 95% CI | | p-value | R^2^/adj. R^2^ |
|  |  |  | Lowest | Highest |  |  |  | Lowest | Highest |  |  |  | Lowest | Highest |  |  |  | Lowest | Highest |  |  |  | Lowest | Highest |  |  |
| Amount of OE (min) during study period; **total sample** |  |  |  |  |  |  |  |  |  |  |  |  |  |  |  |  |  |  |  |  |  |  |  |  |  |  |
|  | 1 | 0.019 | 0.016 | 0.021 | **<0.001** | 0.490 | 0.001 | -0.002 | 0.002 | 0.967 | 0.000 | 0.006 | 0.004 | 0.008 | **<0.001** | 0.149 | 0.003 | 0.000 | 0.005 | **0.032** | 0.023 | 0.005 | 0.003 | 0.007 | **<0.001** | 0.122 |
|  | 2 | 0.020 | 0.017 | 0.022 | **<0.001** | 0.598 | 0.001 | -0.001 | 0.003 | 0.477 | 0.051 | 0.007 | 0.005 | 0.009 | **<0.001** | 0.255 | 0.003 | 0.001 | 0.006 | **0.006** | 0.087 | 0.006 | 0.004 | 0.008 | **<0.001** | 0.235 |
|  | 3 | 0.014 | 0.011 | 0.018 | **<0.001** | 0.641 | -0.002 | -0.005 | 0.001 | 0.158 | 0.087 | 0.003 | 0.001 | 0.006 | **0.015** | 0.321 | -0.000 | -0.003 | 0.003 | 0.996 | 0.142 | 0.002 | 0.000 | 0.005 | 0.064 | 0.313 |
|  | 4 | 0.014 | 0.011 | 0.018 | **<0.001** | 0.636 | -0.002 | -0.005 | 0.001 | 0.235 | 0.077 | 0.003 | 0.001 | 0.006 | **0.015** | 0.308 | 0.001 | -0.003 | 0.004 | 0.697 | 0.138 | 0.003 | 0.000 | 0.005 | **0.044** | 0.306 |
| Interaction term |  | 0.007 | 0.002 | 0.012 | **0.007** | 0.649 | 0.005 | 0.000 | 0.009 | 0.072 | 0.089 | 0.006 | 0.002 | 0.010 | **0.007** | 0.332 | -0.002 | -0.008 | 0.003 | 0.380 | 0.137 | 0.003 | -0.001 | 0.007 | 0.091 | 0.313 |
| Amount of OE (min) during study period; **boys** |  |  |  |  |  |  |  |  |  |  |  |  |  |  |  |  |  |  |  |  |  |  |  |  |  |  |
|  | 1 | 0.016 | 0.011 | 0.021 | **<0.001** | 0.330 |  |  |  |  |  | 0.003 | 0.000 | 0.007 | 0.082 | 0.038 |  |  |  |  |  |  |  |  |  |  |
|  | 2 | 0.016 | 0.011 | 0.021 | **<0.001** | 0.317 |  |  |  |  |  | 0.004 | 0.000 | 0.007 | 0.071 | 0.007 |  |  |  |  |  |  |  |  |  |  |
|  | 3 | 0.001 | 0.004 | 0.018 | **0.003** | 0.337 |  |  |  |  |  | 0.000 | -0.005 | 0.005 | 0.934 | 0.054 |  |  |  |  |  |  |  |  |  |  |
|  | 4 | 0.011 | 0.004 | 0.018 | **0.003** | 0.321 |  |  |  |  |  | 0.000 | -0.005 | 0.005 | 0.962 | 0.023 |  |  |  |  |  |  |  |  |  |  |
| Amount of OE (min) during study period; **girls** |  |  |  |  |  |  |  |  |  |  |  |  |  |  |  |  |  |  |  |  |  |  |  |  |  |  |
|  | 1 | 0.022 | 0.019 | 0.024 | **<0.001** | 0.738 |  |  |  |  |  | 0.008 | 0.006 | 0.011 | **<0.001** | 0.349 |  |  |  |  |  |  |  |  |  |  |
|  | 2 | 0.022 | 0.019 | 0.024 | **<0.001** | 0.743 |  |  |  |  |  | 0.009 | 0.007 | 0.011 | **<0.001** | 0.418 |  |  |  |  |  |  |  |  |  |  |
|  | 3 | 0.016 | 0.013 | 0.019 | **<0.001** | 0.806 |  |  |  |  |  | 0.005 | 0.002 | 0.007 | **<0.001** | 0.512 |  |  |  |  |  |  |  |  |  |  |
|  | 4 | 0.017 | 0.014 | 0.020 | **<0.001** | 0.806 |  |  |  |  |  | 0.005 | 0.003 | 0.008 | **<0.001** | 0.517 |  |  |  |  |  |  |  |  |  |  |
| Amount of OE (min) during study period (ref. 0 min OE); **total sample** |  |  |  |  |  |  |  |  |  |  |  |  |  |  |  |  |  |  |  |  |  |  |  |  |  |  |
| Low to moderate amounts of OE (25–60 min) | 1 | 1.495 | 0.632 | 2.357 | **<0.001** | 0.479 | -0.249 | -0.979 | 0.481 | 0.502 | 0.002 | 0.402 | -0.253 | 1.056 | 0.227 | 0.137 | -0.359 | -1.178 | 0.460 | 0.388 | 0.029 | 0.184 | -0.437 | 0.805 | 0.559 | 0.112 |
|  | 2 | 1.278 | 0.489 | 2.068 | **0.002** | 0.582 | -0.518 | -1.225 | 0.188 | 0.149 | 0.056 | 0.153 | -0.464 | 0.771 | 0.625 | 0.238 | -0.475 | -1.271 | 0.320 | 0.240 | 0.093 | -0.026 | -0.610 | 0.557 | 0.929 | 0.224 |
|  | 3 | 1.278 | 0.466 | 2.090 | **0.002** | 0.632 | -0.506 | -1.266 | 0.254 | 0.191 | 0.091 | 0.165 | -0.478 | 0.808 | 0.613 | 0.312 | -0.265 | -1.114 | 0.584 | 0.539 | 0.140 | 0.042 | -0.562 | 0.647 | 0.891 | 0.306 |
|  | 4 | 1.235 | 0.414 | 2.056 | **0.003** | 0.625 | -0.513 | -1.288 | 0.263 | 0.194 | 0.081 | 0.147 | -0.511 | 0.806 | 0.659 | 0.297 | -0.260 | -1.122 | 0.602 | 0.553 | 0.135 | 0.031 | -0.586 | 0.648 | 0.921 | 0.297 |
| Interaction term |  | 0.682 | -0.828 | 2.191 | 0.374 | 0.636 | -0.131 | -1.572 | 1.311 | 0.858 | 0.090 | 0.147 | -1.063 | 1.350 | 0.811 | 0.318 | 0.828 | -0.783 | 2.438 | 0.312 | 0.134 | 0.342 | -0.807 | 1.490 | 0.558 | 0.302 |
| Highest amounts of OE (120–335 min) | 1 | 5.671 | 4.839 | 6.504 | **<0.001** | 0.479 | -0.092 | -0.797 | 0.613 | 0.797 | 0.002 | 1.778 | 1.146 | 2.409 | **<0.001** | 0.137 | 0.752 | -0.038 | 1.543 | 0.062 | 0.029 | 1.485 | 0.885 | 2.084 | **<0.001** | 0.112 |
|  | 2 | 5.997 | 5.237 | 6.756 | **<0.001** | 0.582 | 0.094 | -0.586 | 0.773 | 0.786 | 0.056 | 2.011 | 1.417 | 2.605 | **<0.001** | 0.238 | 0.939 | 0.173 | 1.704 | **0.016** | 0.093 | 1.705 | 1.143 | 2.266 | **<0.001** | 0.224 |
|  | 3 | 4.302 | 3.329 | 5.276 | **<0.001** | 0.632 | -0.815 | -1.726 | 0.096 | 0.079 | 0.091 | 0.810 | 0.039 | 1.581 | **0.040** | 0.312 | -0.054 | -1.071 | 0.964 | 0.917 | 0.140 | 0.563 | -0.161 | 1.288 | 0.127 | 0.306 |
|  | 4 | 4.239 | 3.234 | 5.243 | **<0.001** | 0.625 | -0.748 | -1.697 | 0.201 | 0.121 | 0.081 | 0.837 | 0.032 | 1.643 | **0.042** | 0.297 | 0.129 | -0.926 | 1.184 | 0.810 | 0.135 | 0.635 | -0.120 | 1.390 | 0.099 | 0.297 |
| Interaction term |  | 2.195 | 0.604 | 3.786 | **0.007** | 0.636 | 1.345 | -0.175 | 2.864 | 0.082 | 0.090 | 1.721 | 0.445 | 2.997 | **0.008** | 0.318 | -0.462 | -2.159 | 1.235 | 0.592 | 0.134 | 1.098 | -0.113 | 2.308 | 0.075 | 0.302 |
| Amount of OE (min) during study period (ref. 0 min OE); **boys** |  |  |  |  |  |  |  |  |  |  |  |  |  |  |  |  |  |  |  |  |  |  |  |  |  |  |
| Highest amounts of OE (120–335 min) | 1 | 4.830 | 3.268 | 6.392 | **<0.001** | 0.327 |  |  |  |  |  | 0.990 | -0.154 | 2.134 | 0.089 | 0.037 |  |  |  |  |  |  |  |  |  |  |
|  | 2 | 4.782 | 3.186 | 6.378 | **<0.001** | 0.305 |  |  |  |  |  | 1.050 | -0.126 | 2.226 | 0.079 | -0.008 |  |  |  |  |  |  |  |  |  |  |
|  | 3 | 3.464 | 1.157 | 5.772 | **0.004** | 0.325 |  |  |  |  |  | -0.151 | -1.829 | 1.526 | 0.858 | 0.047 |  |  |  |  |  |  |  |  |  |  |
|  | 4 | 3.501 | 1.165 | 5.838 | **0.004** | 0.307 |  |  |  |  |  | -0.128 | -1.861 | 1.606 | 0.884 | 0.013 |  |  |  |  |  |  |  |  |  |  |
| Amount of OE (min) during study period (ref. 0 min OE); **girls** |  |  |  |  |  |  |  |  |  |  |  |  |  |  |  |  |  |  |  |  |  |  |  |  |  |  |
| Highest amounts of OE (120–335 min) | 1 | 6.589 | 5.810 | 7.367 | **<0.001** | 0.721 |  |  |  |  |  | 2.428 | 1.740 | 3.106 | **<0.001** | 0.329 |  |  |  |  |  |  |  |  |  |  |
|  | 2 | 6.582 | 5.800 | 7.363 | **<0.001** | 0.720 |  |  |  |  |  | 2.475 | 1.834 | 3.117 | **<0.001** | 0.402 |  |  |  |  |  |  |  |  |  |  |
|  | 3 | 4.695 | 3.831 | 5.558 | **<0.001** | 0.800 |  |  |  |  |  | 1.244 | 0.482 | 2.005 | **0.002** | 0.505 |  |  |  |  |  |  |  |  |  |  |
|  | 4 | 4.815 | 3.901 | 5.728 | **<0.001** | 0.796 |  |  |  |  |  | 1.462 | 0.652 | 2.271 | **<0.001** | 0.508 |  |  |  |  |  |  |  |  |  |  |
| Results from analyses with OE as a continuous and a categorical variable are presented separately. Results are also presented separately for boys and girls if moderator analysis suggested gender differences. Model 1 is unadjusted; Model 2 is adjusted for gender (not included when analysing boys and girls separately), grade and accelerometer wear days; Model 3 is adjusted for gender (not included when analysing boys and girls separately), grade, accelerometer wear days, physical education during study period and season of participation; Model 4 is adjusted for gender (not included when analysing boys and girls separately), grade, accelerometer wear days, physical education during study period, season of participation and socioeconomic status. Missing data were handled using listwise deletion; for each model, participants with complete data on all variables in that specific model were included (n=189–200 [total sample], n=79–81 [boys], and n=110–115 [girls]). R^2^ value is reported for the unadjusted model (1) and adjusted R^2^ value for the adjusted models (3–4). If the interaction term between gender and OE was significant (p<0.05) in the fully adjusted model, the linear regression analyses were conducted separately for boys and girls. Statistically significant results (p<0.05) are in bold. OE = outdoor education, B = Unstandardised regression coefficient, CI = Confidence Interval, R^2^ = Coefficient of determination, min = minutes, min/h = minutes/hour. | | | | | | | | | | | | | | | | | | | | | | | | | | |
