# Supplementary figures and images for: Associations between curriculum-based outdoor education and school-aged children’s physical activity throughout the week
Source: Health Promot Int. 2026 Jul 15;41(4):daag094. doi: 10.1093/heapro/daag094 (PMC13394712; doi:10.1093/heapro/daag094)

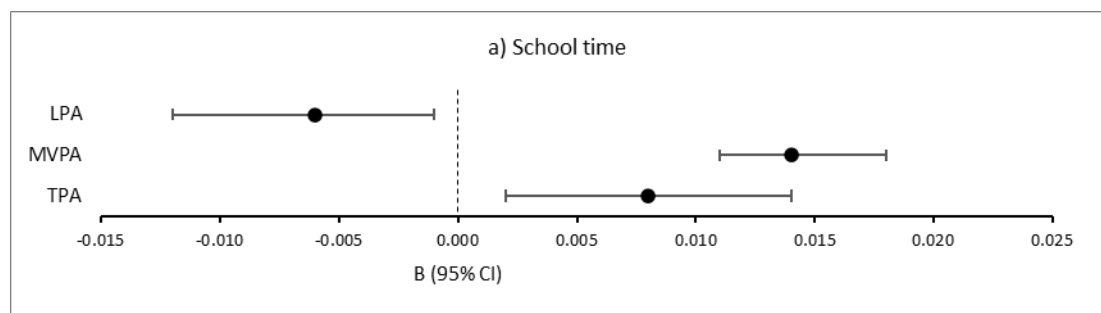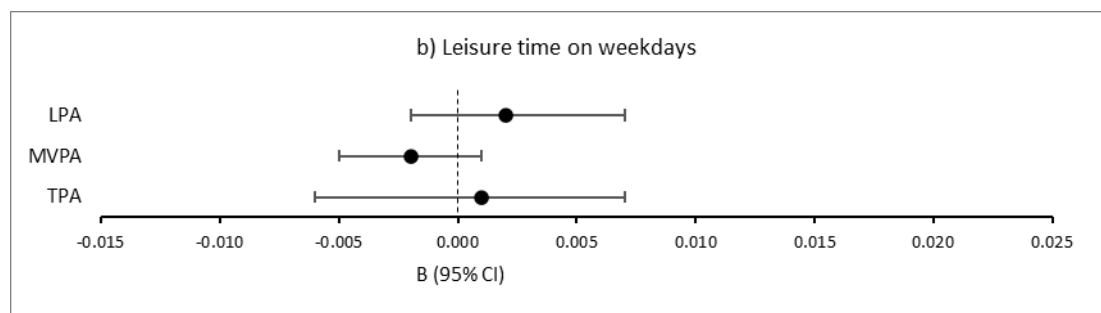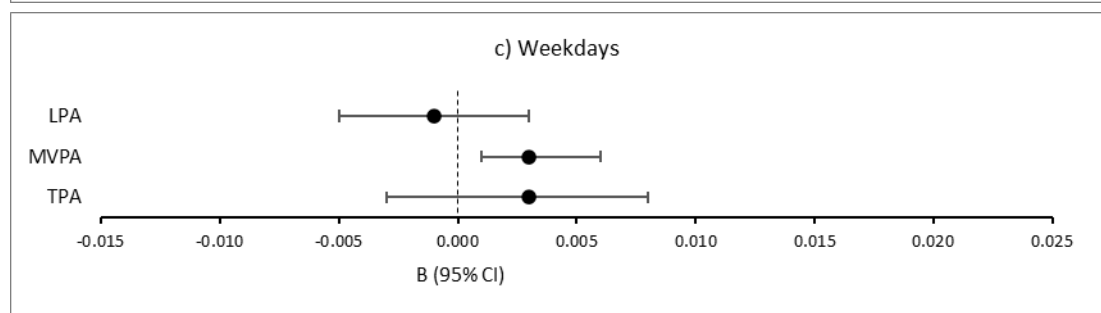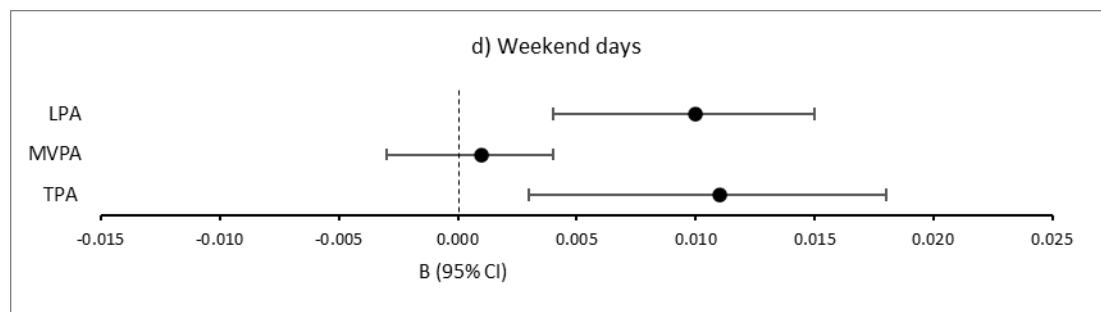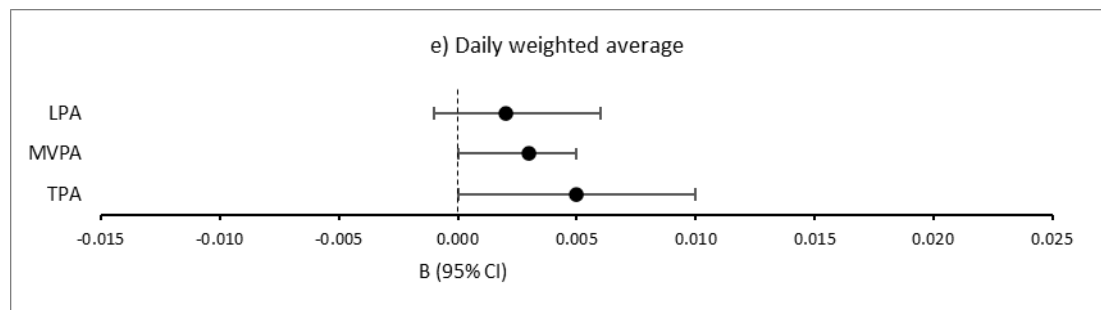

Supplement: daag094_Supplementary_Data [file daag094_supplementary_data.zip › Figure S1.pdf]

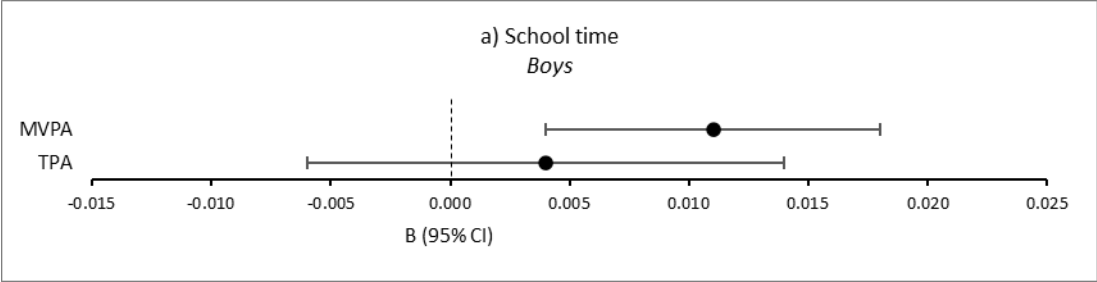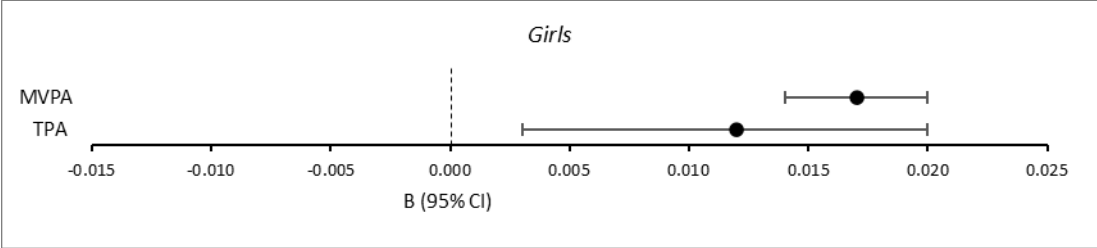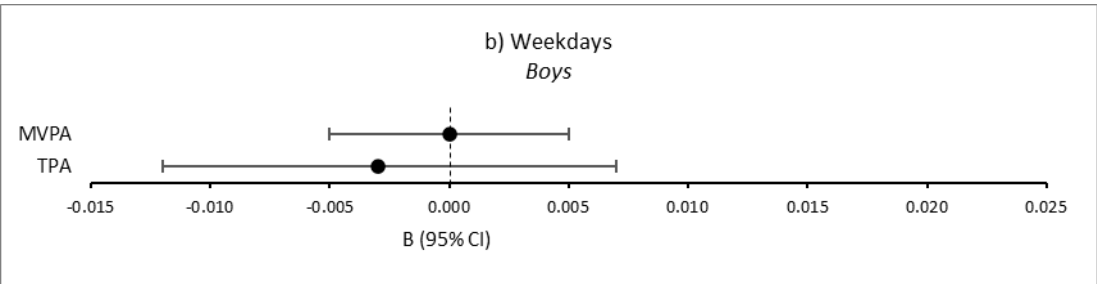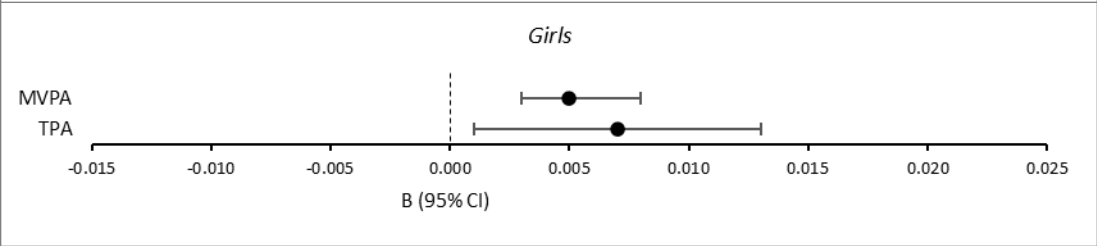

Supplement: daag094_Supplementary_Data [file daag094_supplementary_data.zip › Figure S2.pdf]
